# *Anaplasma phagocytophilum *in Danish sheep: confirmation by DNA sequencing

**DOI:** 10.1186/1751-0147-51-55

**Published:** 2009-12-21

**Authors:** Anne M Kiilerich, Henrik Christensen, Stig M Thamsborg

**Affiliations:** 1 Department of Veterinary Disease Biology, Faculty of Life Sciences, University of Copenhagen, Dyrlægevej 88, DK-1870 Frederiksberg C, Denmark

## Abstract

**Background:**

The presence of *Anaplasma phagocytophilum*, an *Ixodes ricinus *transmitted bacterium, was investigated in two flocks of Danish grazing lambs. Direct PCR detection was performed on DNA extracted from blood and serum with subsequent confirmation by DNA sequencing.

**Methods:**

31 samples obtained from clinically normal lambs in 2000 from Fussingø, Jutland and 12 samples from ten lambs and two ewes from a clinical outbreak at Feddet, Zealand in 2006 were included in the study. Some of the animals from Feddet had shown clinical signs of polyarthritis and general unthriftiness prior to sampling. DNA extraction was optimized from blood and serum and detection achieved by a 16S rRNA targeted PCR with verification of the product by DNA sequencing.

**Results:**

Five DNA extracts were found positive by PCR, including two samples from 2000 and three from 2006. For both series of samples the product was verified as *A. phagocytophilum *by DNA sequencing.

**Conclusions:**

*A. phagocytophilum *was detected by molecular methods for the first time in Danish grazing lambs during the two seasons investigated (2000 and 2006).

## Findings

*Anaplasma phagocytophilum *is the causal agent of granulocytic anaplasmosis (formerly ehrlichiosis) in many animal species as well as in man. The organism holds greatest importance as a cause of tick-borne fever (TBF) or pasture fever in sheep and cattle, but is also a significant pathogen in horses, dogs and cats [1].

The significance of tick-borne infections in Danish livestock is unknown, but outbreaks of *A. phagocytophilum *infection in pastured cattle have been described [2]. The present study was undertaken to examine the occurrence of *A. phagocytophilum *in lambs during summer grazing in Denmark by PCR and DNA sequencing.

Two Danish sheep flocks were investigated. The first flock comprised lambs in a grazing experiment at Fussingø, Jutland in 2000 (Table 1). None of the sampled lambs (or others in the flock) showed signs of clinical illness at the time of sampling. Blood and serum samples were taken by jugular venipuncture from each animal at each sampling time. Blood samples stabilized with EDTA were used for smears and the remainder stored at -20°C. Serum samples were taken in vials with clot-activating factor and after centrifugation frozen immediately at -20°C.

**Table 1:** Data of samples of ovine origin analyzed for *Anaplasma phagocytophilum*

Time	Location of meadows	Flock size	No. of animals sampled	Microscopy Positive	Seropositive	PCR positive		Confirmed by DNA sequencing
							
						Blood	Serum	
June, July and August 2000	Fussingø, Central Jutland	38 lambs	31 samples from 25 lambs	19 samples from 17 lambs	ND	0 (out of 10 samples analysed)	2 (out of 29 samples analysed)	1
								
August 2006	Feddet, Zealand	120 ewes 214 lambs	12 samples from 12 animals (10 lambs and 2 ewes)	0 (only one sample analysed)	1 (only one sample analysed)	3 (out of 12 samples analysed)	ND	2

ND: not determined

Blood samples were examined for *A. phagocytophilum *morula in cytospin white blood cell preparations. In brief, 100 μl EDTA stabilized blood was mixed for 30 sec with 100 μl distilled water for haemolysing red blood cells. Immediately the isotonicity was reestablished by mixing with 100 μl 1.8% NaCl solution. After adding 9.6 ml phosphate buffered saline with 1% bovine serum albumin (PBS-BSA) the sample was mixed and centrifuged for 10 min at 100 × *g*, the supernatant was removed and the cell pellet was resuspended in 400 μl PBS-BSA. A white blood cell preparation was now made on a slide by cytospin preparation at 75 × *g *for 6 min (Shandon Cytospin 2 centrifuge). After drying, the cell preparation was stained with May-Grünwald Giemsa and mounted with Pertex. Four hundred neutrophils were examined for *A. phagocytophilum *in a microscope at 600 × magnification.

Demonstration of neutrophils with free organisms or morula were considered as a probable *A. phagocytophilum *positive case.

The second flock (grazing at Feddet, Zealand) was suspected of a clinical outbreak of TBF and tick pyemia. About 10-20% of the lambs showed signs of polyarthritis and general unthriftiness and several animals were initially treated with amoxicillin. EDTA-stabilized blood samples were taken at random in the flock from ten lambs and two ewes about one and a half month after the major outbreak of clinical signs. Five of the sampled lambs had shown clinical symptoms consistent with TBF at the time of treatment with antibiotics up to two months prior to sampling. Blood samples were kept at -20°C. On one sample from a lamb, fresh blood smear analysis was performed and the serological reaction was determined (indirect fluorescent antibody assay, IFA) by the National Veterinary Institute, Uppsala, Sweden.

Total DNA was extracted from whole blood or serum using the QIAGEN QIAamp DNA Blood Mini Kit based on the manufacturer's instructions (QIAgen, Albertslund, Denmark) with some modifications. According to the manual the theoretical concentration of the eluted DNA should be 15-60 ng/μl (3-12 μg of DNA eluted in 200 μl of buffer AE). Due to low sensitivity in the PCR reactions modified methods for extraction of DNA were tested and in order to concentrate extracts, DNA was eluted in 100 μl of AE buffer supplied by the manufacturer in the final steps of the elution process, which was half of the volume of elution buffer suggested in the manual. Serum samples were centrifuged prior to extraction. As much serum as the size of the sample would allow (up to 1 ml) was centrifuged at 10.000 × *g *for 10 min, and supernatant removed to reduce the volume of the sample to the amount that was to be loaded onto the extraction kit. The pellet was resuspended and DNA of the serum sample extracted.

PCR amplification was performed using the primer pair SSAP2f/SSAP2r [3]. The strongest bands were obtained from blood and serum samples when adding 2 μl and 15 μl of DNA template to PCR reactions, respectively. PCR conditions were initial denaturation at 94°C for 5 min followed by 30 cycles of 94°C denaturation for 30 s, 55°C annealing for 40 s and 72°C extension for 45 s with a final 72°C extension for 7 min followed by cooling at 4°C. The PCR products were analysed on a 1% agarose gel stained with ethidium bromide.

PCR products were purified in MicroSpin™ S-400 HR columns (GE Healthcare) and selected samples sequenced (Macrogen Inc. Seoul, Korea). Sequencing was performed in both directions with the primers used for the initial PCR. Sequences were assembled by Kodon (Applied Maths, Sint-Martens-Latem, Belgium) and compared to published sequences in GenBank [4] by BLAST [5].

The first study in 2000 was carried out in accordance with the requirements of The Danish Animal Ethics Committee. The second study was part of an investigation of a clinical outbreak.

Serum and blood samples from clinically healthy lambs resulted in two PCR positive samples from serum (FS0707 and FS0821). Blood smear analysis was positive for 19 samples (representing 17 animals) spread over the entire sampling period and demonstrated intracytoplasmatic morula or free organisms in neutrophils. The two PCR positive samples represented two different animals, one being positive by blood smear and one negative. Table 1 shows an outline of the number of samples and major results.

Out of 12 blood samples from a flock suspected of an outbreak of TBF and tick pyemia grazing on Feddet, three samples from lambs (I2332, I2333 and I2451) tested positive with the *A. phagocytophilum *specific PCR primer pair SSAP2f/SSAP2r. One of these samples (I2451) was found negative for *A. phagocytophilum *by blood smear analysis, although it was found positive by serology. This sample was the only one to be analysed microscopically and serologically from this flock. Out of these three positive lambs, only one (I2333) had been treated with antibiotics (amoxicillin) prior to sampling.

A partial 16S rRNA gene sequence of 511 bp obtained from the Fussingø sample FS0821 and from the Feddet samples I2451 and I2332 turned out to be identical. BLAST search in GenBank with the sequence obtained in the current investigation showed identity to at least 17 other 16S rRNA gene sequences published for *A. phagocytophilum*. These sequences were obtained from man (CAHU-HGE2, CAHU-HGE1, HZ, USG3), horse, dog and *Ixodes ricinus *but not from sheep. This highest similarity of the sequence in the current study to known sequences of sheep's origin was obtained for *A. ovis *(acc. no. AF318945) with a similarity of 97.6%. Since no *A. phagocytophilum *16S rRNA sequence from sheep was found in the database, the sequence obtained from FS0821 was deposited with acc. no. FJ999757.

By comparison to updated information in GenBank, the primers used for PCR and sequencing could be improved to SSAP2f 5' GCTGAATGTGGGGATTTTTTAT and SSAP2r 5' ATGGCTGCCTCCTTTCGGTTG with suggested changes underlined.

The traditional diagnostic method of *A. phagocytophilum *is microscopic demonstration of the organisms in stained blood smears and serology. Direct visualization is a time consuming method, especially in early stages or in periods of severe leukopenia that follow *A. phagocytophilum *infection. Other purification methods for white blood cells before cytocentrifugation such as Percoll density gradient centrifugation may also be used but are time consuming. Serology by IFA is widely used but may lack in specificity and may not be easily linked to acute disease either due to the lack of antibodies in initial phase of infection or to the presence of residual antibodies resulted from previous infections [6].

PCR with subsequent sequencing of products for confirmation might be more accurate for verification of *A. phagocytophilum *than blood smear counts. Two animals that tested positive by PCR were found negative by blood smear analysis thus indicating false negative detection by blood smear analysis. However, false positive detection needs also to be considered as 17 animals from Fussingø were found positive by blood smear analysis and only one of these was found positive by PCR. The fact that blood smear analysis was performed repeatedly over three months may, however, partly explain a higher detection rate than a single PCR.

PCR alone might lead to false negative detection if the primers are not matching the target or the PCR is not working for other reasons. However, in the case of *A. phagocytophilum *these errors were reduced by inclusion of a positive control and by knowledge of conservation of the 16S rRNA gene sequence used as target for the PCR. The risk of false positives was eliminated by confirmation of the PCR product by sequencing in selected cases. This is needed since the SSAP2 primer pair also amplifies *Ehrlichia canis*. The detection limit for the SSAP2 PCR has not been tested and low levels of *A. phagocytophilum *in the blood might not be detected by the PCR with the risk of false negative results.

The present study demonstrates for the first time the presence of *A. phagocytophilum *in Danish grazing lambs during two seasons on separate geographic locations. A previous investigation has demonstrated *A. phagocytophilum *in Danish ticks by PCR [7].

Limits in access to materials and few samples analysed limited the general conclusions that can be obtained from the present study. For these reasons, more detailed analysis of the epidemiology such as infection rates is not relevant. The microscopic examination as outlined was only performed on samples from year 2000 as we did not find this procedure as accurate as PCR. We suspect that results from blood smear microscopy overestimated the incidence, as 19 blood smears were found positive by microscopy but only one of them was found positive by PCR. A possible explanation for this could be that microscopy was performed by more than one examiner, and that results were not appropriately validated across observers, which could possibly have led to false positives in the judging of samples.

In the first flock (Fussingø) only serum samples and no EDTA-blood samples were found positive by PCR. This could be because the blood samples simply did not come from infected animals, or it could be that factors from the blood and host DNA in the DNA extracts were inhibiting the PCRs. Although it seems contradictory to use serum samples for diagnosing *A. phagocytophilum *infection, as the organism is found intracellularly, serum appears to be a good source of *A. phagocytophilum *DNA, and has been used extensively in other studies [3,8].

In DNA extracts from serum samples less host DNA would be present to interfere with the PCR, and inhibiting factors from the blood, such as heme, would also be absent. In this way it was possible to add a larger amount of DNA extract from serum than from blood to PCR reactions in this study, thereby possibly increasing the total amount of *A. phagocytophilum *DNA in the PCR reaction and thus increasing sensitivity.

Other studies have shown that the buffy-coat fraction can be useful for DNA extraction in PCR testing for *A. phagocytophilum*, as a higher concentration of the leukocytes containing the organism can be obtained [9]. Along with a lower risk of components from the erythrocytes inhibiting the PCR, sensitivity can potentially be increased. However, it was not possible to obtain the buffy-coat fraction from the samples investigated in this study as they had been frozen prior to analysis.

In the first sample flock, no clinical signs were detected that could be referred to *Anaplasma *infection, despite the cumulative incidence of the infection by the end of the grazing season being up to 80% in lambs as determined by blood smear microscopy (data not shown). This is the first time in Denmark that the occurrence of *A. phagocytophilum *has been described in lambs. Due to the mainly subclinical and self limiting course [1] the infection may be widespread in animals grazing *I. ricinus *habitats without notice [7]. Preliminary results have shown that the prevalence of *A. phagocytophilum *in roe deer in Denmark is widespread covering almost all parts of the country [10] but further epidemiologic studies are needed to establish the distribution of *A. phagocytophilum *in domestic animals.

Surprisingly, none of the 16S rRNA gene sequences from *A. phagocytophilum *obtained in GenBank from sheep showed identity to the sequences isolated in this study. It needs to be investigated if different populations of *A. phagocytophilum *might show host associations. The use of 16S rRNA gene sequence comparison offers limited or no resolution at the species level and other techniques such as multilocus sequences typing would be required for such kinds of investigations.

## Competing interests

The authors declare that they have no competing interests.

## Authors' contributions

AMK carried out the molecular genetic studies, participated in the sequence alignment and drafted the manuscript. HC participated in the sequence comparison and in writing of the manuscript. SMT provided sample material, participated in the design of the study and coordinated and helped to draft the manuscript. All authors read and approved the final manuscript.
